# Multiple vas deferens with polyorchidism and many congenital malformations in a symptomatic 11-year-old male patient: a rare case report

**DOI:** 10.1186/s12894-022-00972-2

**Published:** 2022-02-23

**Authors:** Marah Mansour, Mohammad Adel Ismail, Mohammad Ali Dashan, Ahmad Kheat, Tamim Alsuliman, Khaled Alrebdawi

**Affiliations:** 1 Faculty of Medicine, Tartous University, Tartous, Syrian Arab Republic; 2 Faculty of Medicine, University of Aleppo, Aleppo, Syrian Arab Republic; 3 Department of Cardiology, Lattakia National Hospital, Lattakia, Syrian Arab Republic; 4 Hematology and Cell Therapy Department, Saint-Antoine Hospital, AP-HP, Sorbonne University, Paris, France; 5 Department of Urological Surgery, Surgical Kidney Hospital, Damascus, Syrian Arab Republic

**Keywords:** Duplication, Renal dysplasia, Cystourethroscopy catheterization, Retrograde urethrogram, Vasography, Resection, Case report

## Abstract

**Background:**

Ductus deferens may manifest in a variety of anomalies such as its absence, duplication, ectopy, or diverticulum. Ectopic seminal tract opening has two main types, ectopic ejaculatory duct opening, and ectopic vas deferens opening. Generally, ductus deferens anomalies affect approximately 0.05% of the population. Patients may be asymptomatic or complaining of urinary tract infections and/or epididymitis. Most of these cases are associated with renal dysplasia. To confirm the diagnosis Cystourethroscopy catheterization and retrograde urethrogram should be performed, but the definitive diagnosis is done by vasography. The definitive treatment is complete surgical resection of the pathological urogenital connection. This case is commonly discovered while exploring other findings such as testicular torsion and inguinal hernia.

**Case presentation:**

We report a rare case of an 11-year-old male who presented with gross hematuria and numerous congenital malformations including a left polydactyly clubfoot, polyorchidism, with several surgical procedures, and left kidney dysgenesis. Surgery was performed for a left inguinal hernia, during which a third undescended testicle was discovered incidentally and was eradicated. A retrograde urethrogram was performed to establish the diagnosis. A fistula- that is connected with the left ureter- was resected. The histopathologic findings confirmed the diagnosis of true duplication of the Vas deferens, with communication between the ureter and the vas deferens. By follow-up, the kidney function tests were within normal limits.

**Conclusions:**

This case report aims to highlight the early diagnosis and management of the duplicated vas deferens and the associated congenital malformations to improve the prognosis and kidney function and to avoid long-term complications.

## Background

The congenital anomalies of the ductus deferens, which are rare, affect approximately 0.05% of the general population. This phenomenon has two main types, ectopic ejaculatory duct opening, and ectopic vas deferens opening [1]. In general, Ductus deferens' precise etiology is still unknown. Patients may be asymptomatic or complaining of urinary tract infections and/or epididymitis [2]. The insertion of Ductus deferens into the Ureter is a rare congenital malformation. Most of these cases are associated with renal dysplasia [2]. The diagnosis should be confirmed by Cystourethroscopy catheterization and retrograde urethrogram, but the definitive diagnosis is done by vasography [2]. Curative management is preferred to be complete surgical severance of the pathological urogenital connection [3]. Whereas, Triorchidism-the most common form of polyorchidism-is the presence of three testicles. This case is commonly discovered while exploring other findings such as testicular torsion and inguinal hernia [4]. In this case, we report an 11-year-old male with polydactyly, polyorchidism, and dysplastic kidney.

## Case presentation

**Fig. 1:**
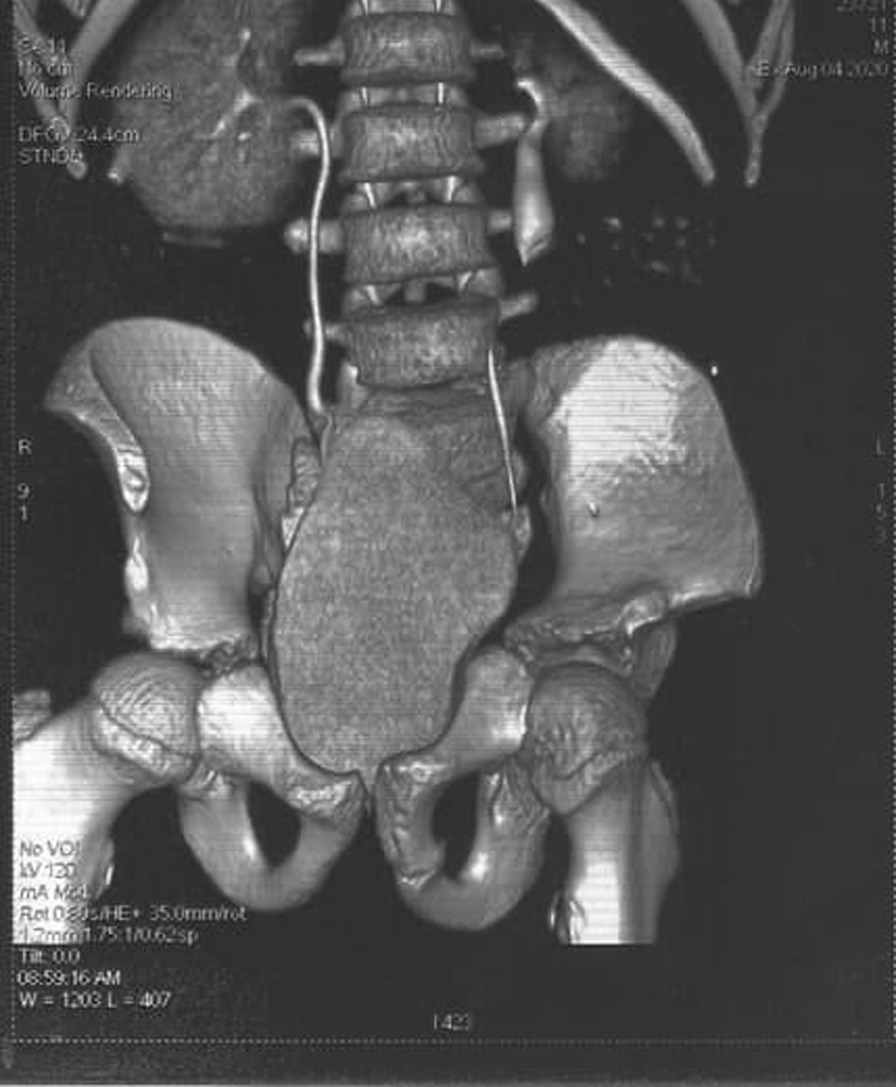
CECT showing atrophy in the left kidney along with dilation of the left ureter

**Fig. 2:**
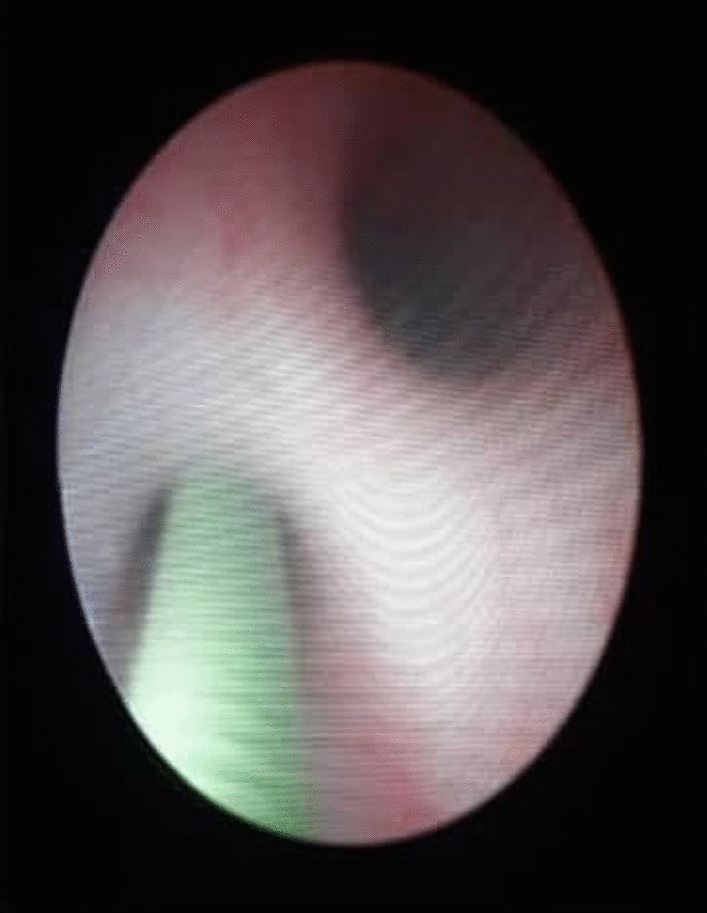
Left ureteroscopy demonstrating additional meatus

**Fig. 3:**
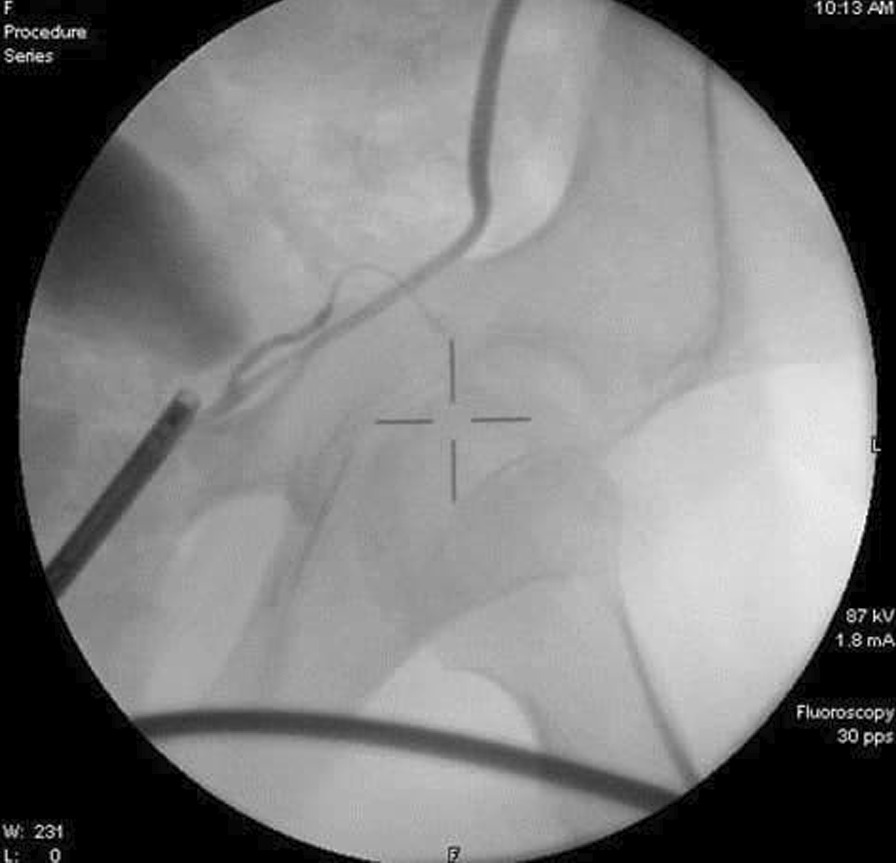
Dye injection before bifurcation, showing dilated both the ureter and the fistula

**Fig. 4:**
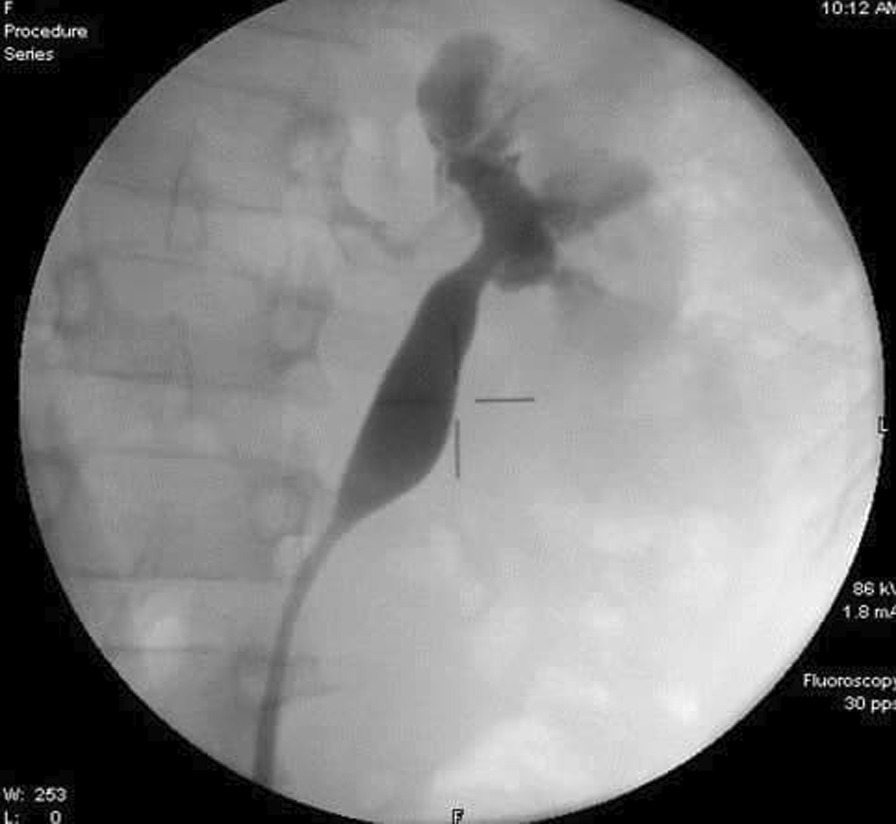
Dye injection in the left ureter, showing the pelvis and the ureter

**Fig. 5:**
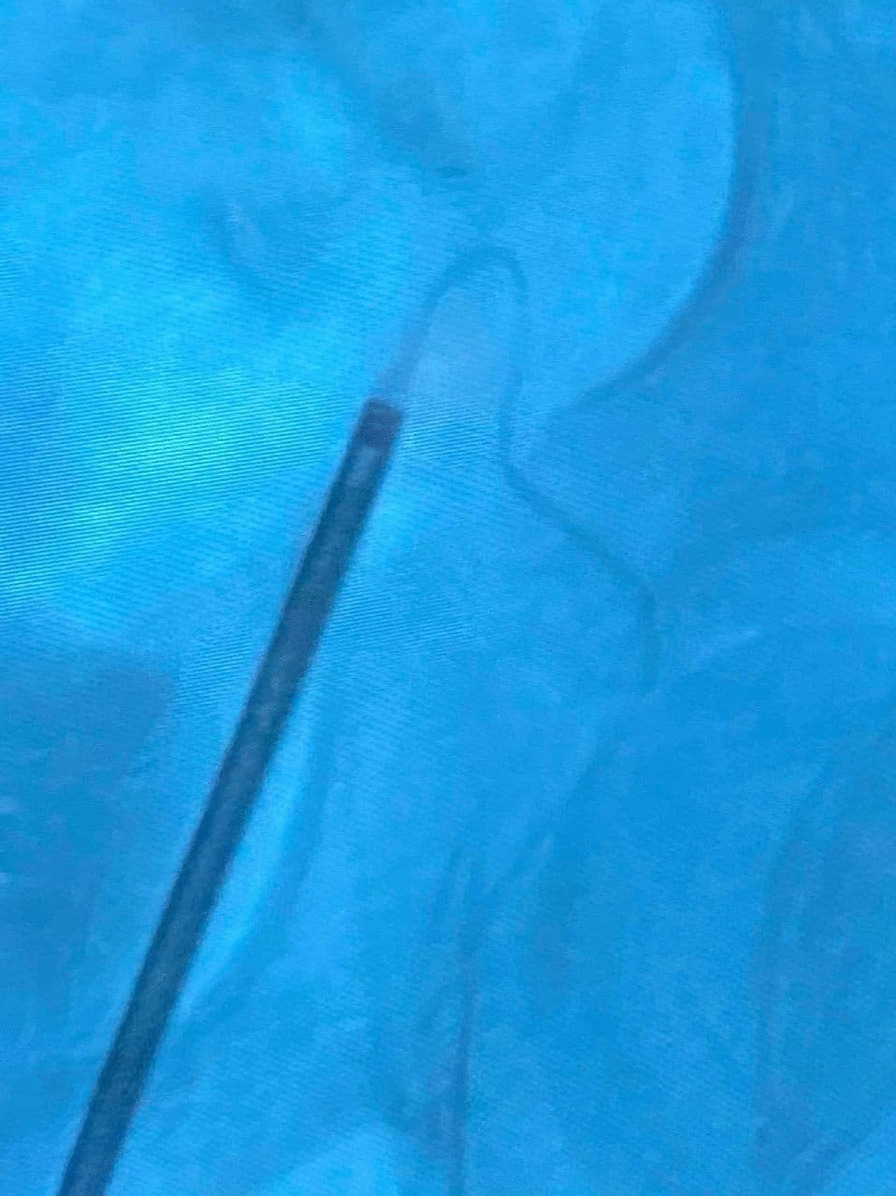
Dye injection into the fistula down to the left scrotal sac

**Fig. 6:**
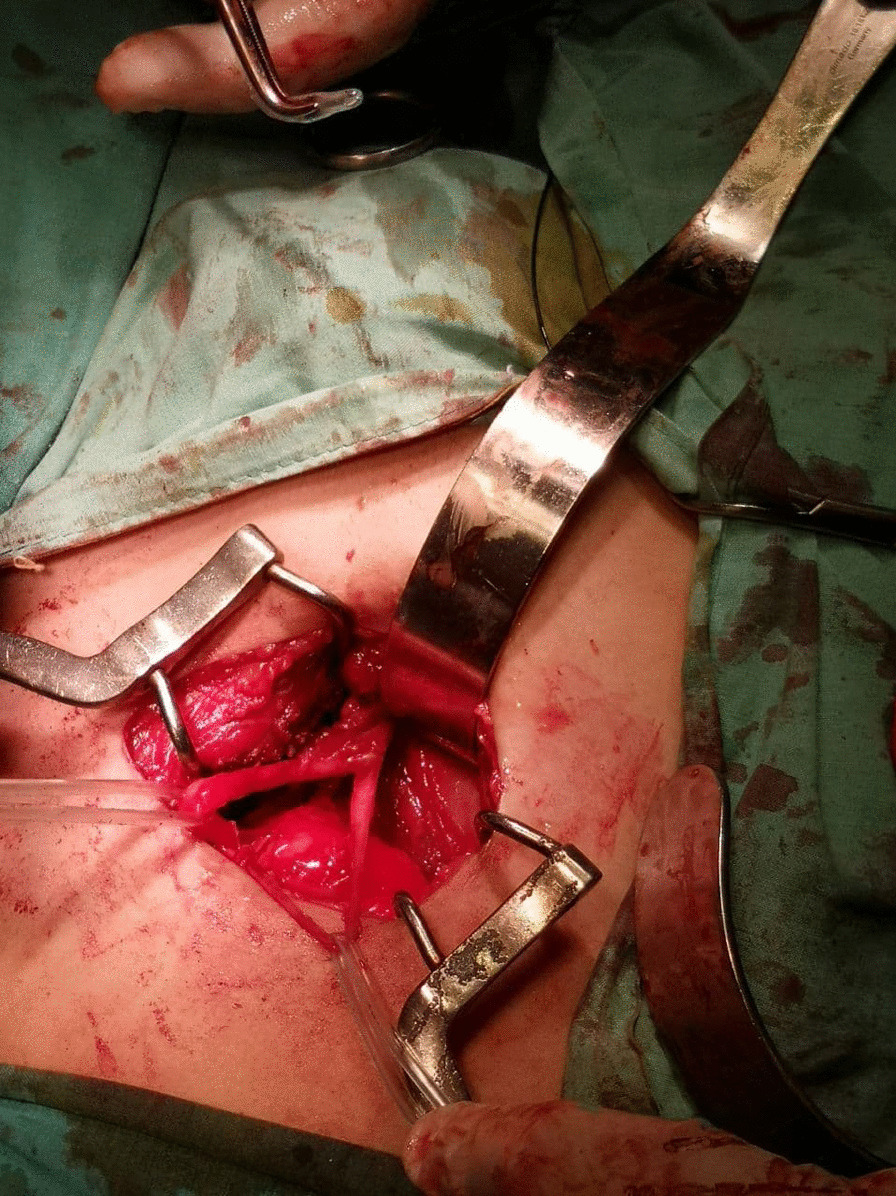
The left ureter is to the left and the ectopic ductus deferens is to the right of the image

An 11-year-old male presented to the Department of Urology with gross hematuria in the last few days. The medical history included numerous congenital malformations: a left polydactyly clubfoot with several surgical procedures and left kidney dysgenesis. Surgery was performed for a left inguinal hernia at the age of 1.5 years, during which a third undescended testicle was discovered incidentally and was eradicated without the vas deferens that were only ligated. There was no oliguria, dysuria, or any significant symptoms. Physical examination and laboratory findings were within normal limits, excepting hyperphosphatemia (7.3 mg/dl). A sample urine test demonstrated increased protein and creatinine in urine (84.6 mg/dl) and (380 mg/dl) respectively. A urinalysis result revealed 72 RBC (per cubic mm), 95% of them isomorphic (lower urinary tract origin). Furthermore, no schistosome eggs were observed in the urine. Abdominal Contrast-Enhanced Computed Tomography (CECT) demonstrated important details that the left kidney was relatively smaller with diagonals (5 × 3 cm), (8 mm) cortical thickening, and atrophy in its artery (diagonal 1.6 mm). Moreover, a mild dilation in the upper half of the left ureter was discovered without hydronephrosis along with scoliosis in the lower and sacral part of the vertebral column along with malformation in the pelvis form (Fig. 1). Afterward, a ureteroscope was done for the left ureter and another meatus was discovered (Fig. 2). Thus, retrograde uretero-pyelography was performed using a fiberoptic ureteroscope. During cystoscopy, contrast dye is injected into the ureter. Firstly, the dye was injected before the second meatus, and both the ureter and the fistula were shown (Fig. 3). Secondly, the dye was injected separately in the left ureter after the bifurcation and in the fistula (Figs. 4, 5). Consequently, the patient underwent a second surgery. A fistula- that is connected with the left ureter- was resected (Fig. 6). The histopathologic findings confirmed the diagnosis of Vas deferens, with no evidence of malignancy. After a three-month follow-up, the patient had normal kidney function tests with no more hematuria.

## Discussion and conclusions

The ectopic seminal tract opening was first reported in 1895. There is a variety of ductus deferens anomalies that could be observed such as absence, duplication, ectopy, and diverticulum. The duplication of ductus deferens differs from the double vas deferens. The first term refers to the presence of two separated ductus deferens within the spermatic cord, whereas the latter is described with an ectopic ureter draining into the ejacularity system [5, 6]. Embryologically, the formation of polyorchidisim happens when longitude or transverse division occurs in the mesonephric duct. When the duplication occurs at the superior end of the mesonephric duct, the polyorchidisim associated with duplicated ductus deferens [4]. In addition, the embryological reason for the insertion of the ductus deferens into the ureter is the failure of obliteration of the potion of the Wolffian duct that receives both the ureter and the ductus deferens, which results in ectopy ductus deferens into the ureter [2]. Histologically, the specimen shows a tubal structure having a thick muscle coat and lined by almost completely eroded epithelium with edema and inflammation in the subepithelial connective tissue. However, there is a tiny residual part of the epithelium composed of stratified columnar epithelium. The overall histologic composition of this structure is consistent with vas deferens, with no evidence of malignancy. Liang has proposed a classification system for duplicated ductus deferens, which includes 3 types of duplication: Type1: Duplicated’’ vas deferens in spermatic cord with no polyorchidism, Type II: Multiple vas deferens with polyorchidis, and Type III: False poly-vasa deferential including ‘‘double’’ vas deferens, ureter draining into the ejaculatory system [6]. Subsequently, our case is considered as type II associated with other malformations: a left polydactyly, clubfoot, and left kidney dysgenesis. Ductus deferens duplication is most commonly noticed during orchiopexy, inguinal hernia, vasectomy, varicocelectomy, and radical prostatectomy [5, 6]. The clinical presentation of this case includes anus atresia, hypospadias with undescended testes, infertility, swollen scrotum, hematuria, and urinary tract infection [7]. The retrograde passage of urine into the vas deferens can cause epididymitis and even scrotal abscesses when associated with hydroureter because of vesicoureteral reflux or obstruction [2]. Radiological study of the bladder and ureters by retrograde uretro-pyelography is the procedure most likely to confirm the diagnosis. In this method, the contrast medium refluxes up the ureter and passes into the vas, which is the method we used in this case. Other investigations such as computed tomography (CT) scan can be performed to confirm other abnormalities in the genitourinary tract if a duplicated vas deferens is found during surgery. Doppler's sonography can be also important and useful to differentiate duplicated vasa deferentia from spermatic vessels. VCUG was done and turned out to be normal. Unfortunately, laparoscopy was not applied as a result of war-torn health services and the shortage of advanced tools. The curative procedure of the fusion between the ureter and the vas deferens is confirmed to be surgical severance [8]. About surgery, Morrison’s incision, deepening to the back of the peritoneum, isolation of the ureter where it connects with the ductal deferens, cutting the ductal deferens where it connects with the ureter with its excision, placing a DJ in the ureter, closing the ureter, placing a detonator and closing the wound. The aim of the management of patients with additional ductus deferens draining into the ureter is to prevent Urinary Tract Infection because of vesicovasalor viscoureter vasal reflex [2], and to avoid iatrogenic injury, failure of vasectomy for male contraception, and to exclude associated renal anomalies [6]. In this case, the surgical procedure was performed to manage the duplicated vas deferens and its manifestations and to manage other abnormalities associated with this phenomenon, such as polydactyly surgery. The follow-up showed a very good prognosis and kidney function with no evidence of recurrence or abnormality.

## Data Availability

Not applicable. All data (of the patient) generated during this study are included in this published article and its supplementary information files.

## Abbreviations

CECT: Contrast-enhanced computed tomography
CT: Computed tomography
VCUG: Voiding cystourethrogram
